# Response to – Counteracting white matter injury to mitigate APOE4‐related ARIA

**DOI:** 10.1002/alz.71095

**Published:** 2026-01-28

**Authors:** Zahra Shirzadi, Jasmeer P. Chhatwal

**Affiliations:** ^1^ Department of Neurology, Harvard Medical School Brigham and Women's Hospital Cambridge Massachusetts USA; ^2^ Department of Neurology, Harvard Medical School Massachusetts General Hospital Boston Massachusetts USA

**Keywords:** ARIA, cerebral amyloid angiopathy, vascular risk factors, White matter hyperintensity

1

We thank the author of this letter for the careful reading of our research article and appreciate the thoughtful points raised. We agree that white matter hyperintensity (WMH) is an important consideration in the appearance of amyloid‐related imaging abnormalities (ARIA), particularly ARIA‐H, as highlighted in this paper.^1^ We believe that this association may be primarily driven by latent, “silent” cerebral amyloid angiopathy (CAA) that has not yet manifested in magnetic resonance imaging (MRI)‐visible microhemorrhages, and which may be exacerbated by the administration of anti‐amyloid antibody therapies.

We would like to take this opportunity to emphasize the multifactorial nature of WMH, as demonstrated in several studies by our group and others.^2^, ^3^, ^4^ In the A4 dataset specifically, we observed that WMH volume and its rate of progression were independently associated with older age, CAA, gray matter atrophy, and systemic vascular risk.^2^ These findings underscore that WMH is not a singular phenomenon but rather reflects a complex interplay of CAA, neurodegenerative, and vascular processes.

In this context, we concur with the author that management of systemic vascular risk—including elevated blood pressure, diabetes, and other modifiable factors—should be prioritized in individuals at risk for Alzheimer's disease (AD). Such interventions may not only help reduce WMH burden but could also potentially slow AD progression.^5^, ^6^, ^7^, ^8^ This perspective aligns with growing evidence that vascular health is a critical component of brain aging and neurodegenerative disease prevention.

Finally, we agree that further research is warranted to examine whether aggressive control of systemic vascular risk factors can mitigate the emergence of ARIA in patients receiving amyloid‐targeting treatments. Understanding this interaction will be essential for optimizing therapeutic strategies and improving safety profiles for individuals undergoing disease‐modifying interventions.

## CONFLICT OF INTEREST STATEMENT

The authors declare no conflicts of interest.

## Supporting information

Supporting Information
